# Stress and depressive symptoms in university students in Hong Kong under the pandemic: Moderating effect of positive psychological attributes

**DOI:** 10.3389/fpsyg.2023.1071938

**Published:** 2023-01-26

**Authors:** Daniel T. L. Shek, Wen-yu Chai, Tingyin Wong, Kaiji Zhou

**Affiliations:** Department of Applied Social Sciences, The Hong Kong Polytechnic University, Kowloon, Hong Kong SAR, China

**Keywords:** positive psychological factors, COVID-19, stress, depression, moderating effect, Chinese university students

## Abstract

**Introduction:**

There are very few studies examining the psychological well-being of university students in Hong Kong under the COVID-19 pandemic. Besides COVID-19-related stress, the “social event” in 2019-2020 has caused significant stress in young people. As such, we attempted to answer several research questions in this paper. First, what are the relationships between stresses (COVID-19 and “social event” related stresses) and psychological well-being indexed by depressive symptoms? Based on the stress and coping perspective, we predicted that there would be positive relationships between both types of stress and depression. Second, what are the relationships between different positive psychological factors (including life satisfaction, resilience and emotional management, flourishing, and beliefs about adversity) and depression? Based on different theoretical models of positive psychology, we hypothesized that negative relationships would exist between positive psychological factors and depressive symptoms. Third, do positive psychological attributes moderate the associations between stresses (COVID-19 and “social event” related stresses) and depressive symptoms? Based on the positive psychology literature, we hypothesized that positive psychological attributes would buffer the negative impact of stresses on depression.

**Methods:**

We recruited university students roughly one year after the first wave of the pandemic (*N* = 1,648) in early 2021. We used 25 items to measure COVID-19-related stress and “social event” related stress. For psychological well-being indexed by depressive symptoms, we used the “Centre for Epidemiologic Studies Depression Scale Revised (CESD-R)”. For positive psychological attributes, we employed established measures of life satisfaction, resilience and emotional management, flourishing, and beliefs about adversity.

**Results:**

Regarding the relationship between stress and depression, we found positive relationships between both types of stress and depressive symptoms. As predicted, negative relationships existed between all positive psychological attributes and depressive symptoms. Besides, the positive psychological attributes significantly moderated the effects of stresses on depression, suggesting that these factors can reduce the negative impacts of stresses on depression. The present findings provide support for those models, highlighting the importance of positive psychological attributes as protective factors for university students’ depression.

**Discussion:**

The findings of this study underscore the important role of positive psychological attributes in the stress-depression relationship in university students under the pandemic. The findings also generalize the positive youth development theory in the Chinese context. In terms of practice, university administrators and service providers should consider cultivating positive psychological attributes in university students with the purpose of promoting their psychological well-being.

## 1. Introduction

The 2019 coronavirus disease (COVID-19) pandemic has raised significant challenges to people all over the world. Particularly, university students face significant challenges and difficulties under the pandemic due to the lockdown policies, school closure, and drastic change from face-to-face learning to online learning (Nassr et al., 2020; Schiff et al., 2021). All these challenges, together with worries about the disease, would cause high stress in college students and further harm their mental health, such as leading to depressive symptoms. However, there are very few studies on the association between pandemic-related stress and depression in university students, as well as the protective factors involved. With particular reference to Hong Kong, besides stress arising from the COVID-19, the “social event” in 2019–2020 is also a significant stressor for people in Hong Kong (Shek, 2020). Hence, it is necessary to understand the associations between both stresses (i.e., COVID-19 and “social event” related stresses) and mental health in university students. In view of this research gap, this study examined the relationships between stresses from the pandemic and “social event” and mental health (indexed by depression) as well as the moderating effects of positive psychological factors on the stress-mental health relationships in Hong Kong university students.

## 2. Literature review

### 2.1. COVID-19 and “social event” related stresses and depression

Literature shows that COVID-19 stress is positively related to depressive symptoms among different samples of populations and in adolescents and college students. In US adults, Ettman et al. (2020) found that low social and economic resources and high exposure to stressors during the pandemic were associated with a higher risk of depression. In the general population in the Philippines, Tee et al. (2020) revealed that 16.3% of their research participants rated moderate-to-severe psychological impact from the COVID-19 outbreak and 16.9% of the participants showed moderate-to-severe levels of depression. In adolescents, Ellis et al. (2020) reported that COVID-19 stress was associated with increased loneliness and depressive symptoms. In college students, Lu et al. (2022) found that COVID-19 stress was positively associated with symptoms of depression. Galanza et al. (2021) showed that COVID-19-related fear predicted three mental illness indicators, including depression, stress, and anxiety. In addition, Rudenstine et al. (2021) revealed that increased exposure to pandemic-related stressors predicted symptoms of depression and anxiety.

Besides adolescents and university students, studies also showed that pandemic stress was related to mental health, particularly depression, in other groups of people. Filippetti et al. (2022) and Lebel et al. (2020) revealed that COVID-19 stress was positively associated with symptoms of anxiety and depression in pregnant women. Fong et al. (2022) found that high levels of perceived COVID-19 impact, perceived stress, anxiety, and caregiving burden were associated with probable depression of caregivers of persons with dementia. However, in emerging adults, Kujawa et al. (2020) examined the relationship between perceived COVID-19-related stress and mental distress over time and found that baseline stress did not predict changes in anxiety and depressive symptoms at Wave 2, suggesting that COVID-19 stress may not have a long-term influence on individuals’ mental health.

In the Hong Kong context, there are studies investigating the influence of both COVID-19-related stress and social-unrest-related stress on the psychological health of Hong Kong people. Wong et al. (2021) examined the impact of “social-unrest-related traumatic events (TEs),” “pandemic-related events (PEs),” and “personal stressful life events (SLEs)” on individual depressive and post-traumatic stress disorder (PTSD) symptoms. The study showed that TEs, SLEs, and rumination predicted PTSD symptoms, while PEs, SLEs, and rumination predicted depressive symptoms. Hou W. K. et al. (2021) studied the joint effect of COVID-19 and the social unrest on Chinese residents’ anxiety and depressive symptoms between February and May 2020. They found strong associations between stresses arising from both the social unrest and COVID-19 and symptoms of anxiety and depression. Based on four waves of data collected from July 2019 to August 2020, Hou et al. (2022) also revealed that stresses related to both the social unrest and the pandemic were associated with probable anxiety and depression. While these studies examined the effects of both social unrest stress and COVID-19 stress on depression, they mainly focused on community and adult samples but not university student samples. In addition, these studies did not examine the moderating effect of positive psychological attributes in the relationships between stress and mental health. As university students are a highly vulnerable group under the pandemic (e.g., school closure) and social movement (e.g., physical or psychological involvement), it is important and necessary to examine the predictive effects of stresses arising from both COVID-19 and the “social event” on depression in Hong Kong university students. In addition, it is important to understand the protective role of positive psychological attributes in the relationships.

### 2.2. Positive psychological factors and depression

Positive psychology stresses that psychology should not only focus on mental problems because strengths and positive qualities in human beings are important (Seligman and Csikszentmihalyi, 2000). Scholars promoting positive psychology also argue that psychological strengths are essential in promoting human well-being and in prevention of illness (Gable and Haidt, 2005). Conceptually, beliefs about adversity (including hope and optimism), psychosocial competence (resilience and emotional competence), flourishing, and life satisfaction are foundational elements in positive psychology (e.g., Seligman and Csikszentmihalyi, 2000; Yates et al., 2015; Compton and Hoffman, 2019). Typically, life satisfaction is regarded as an important adolescent developmental outcome measure. However, it is also regarded by researchers in positive psychology as a factor that predicts, mediates, or moderates the relationship between adolescent experience and well-being (Gilman and Huebner, 2003; Huebner et al., 2006). There are studies showing that life satisfaction is a mediator shaping psychological well-being outcomes (Shek and Li, 2016; Lopez-Zafra et al., 2019; Zhou et al., 2020). Therefore, these four attributes were adopted as indicators of positive psychological attributes in the present study.

#### 2.2.1. Beliefs about adversity and depression under the pandemic

Previous studies showed that beliefs about adversity, such as hope and optimism, are associated with lower levels of depression under the pandemic (Shek et al., 2022a,b). Marciano et al. (2022) reported that hope had a stronger and more consistent predictive power than did health, economic, security, and political threats on individuals’ well-being, including depressive symptoms. Schug et al. (2021) reported that social support and optimism had associations with low levels of depression and generalized anxiety. In addition, scholars found a negative association between trait optimism and depressive symptoms amid the pandemic. Dillard et al. (2022) reported that higher trait optimism was related to lower levels of perceived pandemic-related impact and depressive symptoms in women. Riepenhausen et al. (2022) observed that trait optimism significantly predicted changes in depressive and anxiety symptoms in German people before and during the pandemic.

Researchers have also reported the moderating role of hope and optimism in the relationship between COVID-19 stress and depression. For example, Haddad et al. (2021) showed that optimism could reduce the negative effect of problematic social media use on mental health during the pandemic. Hou L. L. et al. (2021) also reported that dispositional optimism moderated the influences of quarantine length on worry and anticipation on depression. Goh et al. (2022) found that hope moderated the relationship between job loss and depressive symptoms of mothers in financially disadvantaged families during the pandemic. On the other hand, Kuhlman et al. (2021) did not identify evidence for the moderating function of optimism in relationship between COVID-19-related impact (pandemic-related concern and events) and psychosocial outcomes (depression, anxiety, aggression, and sleep quality).

#### 2.2.2. Psychosocial competence (resilience and emotional competence) under the pandemic

Studies based on different populations generally suggested that resilience was negatively related to depression amid the COVID-19 pandemic. Using data collected through social media, Karasar and Canli (2020) found that psychological resilience was negatively correlated with depression. Zhang et al. (2020) revealed that resilience was inversely associated with psychological morbidity and could act as a protective factor against depression and anxiety in patients with mild COVID-19 symptoms in Wuhan, China. Matsumoto et al. (2022) showed that low psychological resilience was related to depression in elderly with mild cognitive impairment under the pandemic. In addition, Schmitt et al. (2021) found that Brazilian participants with higher levels of resilience showed lower levels of depression.

Studies also showed that resilience moderated the association between pandemic stressors and depression. Chan et al. (2021) showed that stronger individual resilience reduced the adverse effect of pandemic stressors on depressive symptoms. Vos et al. (2021) revealed that COVID-19-related fear was positively linked with depression, stress, and anxiety, while the adverse effect on mental health was reduced by high levels of resilience, mindfulness, and optimism. Yildirim et al. (2022) also showed that perceived risk and COVID-19-related fear could positively predict depression, stress, and anxiety, but resilience reduced the effect of pandemic-related fear on mental distress. However, some other studies have failed to detect the moderating effect of resilience in the COVID-19 stress-depression relationship (e.g., Traunmuller et al., 2021).

#### 2.2.3. Flourishing and depression under the pandemic

Past research has shown the protective role of flourishing in the mental health of different groups of people under the pandemic. For children, Jang et al. (2020) revealed that flourishing negatively predicted children’s levels of depression. Liu and Wang (2021) examined perceived stress of COVID-19, character strengths (a measure of flourishing), and depression in adolescents. They found that character strengths moderated the influence of pandemic-related stress on depressive symptoms. Based on longitudinal data collected from three cohorts of adults, Burns et al. (2022) concluded that, together with moderate well-being, “flourishing conferred protection against any depression and mental health symptoms in all age cohorts in comparison with languishers (i.e., those with low well-being)” (p. 6). Investigating workers’ mental health during the pandemic, Mendonca et al. (2022) also showed that flourishing played a protective role against mental distress (e.g., depression, stress, and anxiety) in different work situations. Despite a number of existing studies in this field, the direct evidence of the protective effect of flourishing against depression among university students during the pandemic is rare.

#### 2.2.4. Life satisfaction and depression under the pandemic

Studies generally showed that life satisfaction was negatively associated with depression. In adolescents, Kaya and McCabe (2022) reported that adolescents with higher-level depression had higher-level loneliness and lower-level life satisfaction. In Brazilian college students, Lopes and Nihei (2021) also showed a negative relation between depressive symptoms and life satisfaction, suggesting that life satisfaction could act as a protective factor for students’ mental health. Based on adults working in nursing home during the pandemic, Navarro-Prados et al. (2022) showed that resilience, satisfaction with life, and personal accomplishment related negatively with depression and emotional exhaustion.

Besides a direct relationship between life satisfaction and depression, studies also showed that life satisfaction mediated the association between COVID-19 stresses and depression. For example, Tamarit et al. (2022) revealed that resilience and life satisfaction mediated the association between pandemic-related worries and negative emotional symptoms (depression, anxiety, and stress). Coello et al. (2022) also showed that life satisfaction had a mediating role in the relationship between pandemic-related worries and depression, anxiety, and stress. Satici et al. (2021) showed that depressive symptoms mediated the link between fear of the pandemic and life satisfaction. Interestingly, there are very few studies examining the moderating function of life satisfaction in the COVID-19 stress-mental health association.

## 3. The present study

Based on the review of existing literature, there are several research gaps. First, as many studies focused on adults or other groups of people, there was inadequate research on adolescents and university students. Second, as many existing studies are Western studies, there was inadequate research conducted in non-Western contexts. Third, with regard to the moderating function of different positive psychological factors such as resilience, there are inconclusive findings. Fourth, there are few studies investigating the predictive effect of both stresses (i.e., stresses related to COVID-19 and the “social event”) on depression. In view of the research gap, this study attempted to investigate the predictive role of stresses from the pandemic and the “social event” in depression in Chinese university students and the moderating role of positive psychological factors. Specifically, in this study, we asked three research questions:

Research Question 1: What are the associations between stresses arising from COVID-19 and from the “social event” and depression? According to the literature, we hypothesized that both types of stresses would be positively associated with depression (Hypotheses 1a and 1b).
Research Question 2: What are the associations between positive psychological attributes (indexed by life satisfaction, flourishing, beliefs of adversity, and resilience and emotional competence) and depression? The above discussion suggests that negative associations would exist between positive psychological attributes and depression (Hypothesis 2).
Research Question 3: Do positive psychological attributes moderate the negative impacts of stresses from COVID-19 and from the “social event” on depression? Based on the literature on protective factors, we expected that under lower levels of positive psychological attributes, the linkage between both stresses and depression would be stronger than the linkage under higher levels of positive psychological attributes (Hypotheses 3a and 3b).

## 4. Methods

### 4.1. Participants and procedure

Between January and March 2021, students from undergraduate programs in one public university in Hong Kong were invited to do an online survey questionnaire through Qualtrics XM (an online survey platform; Shek et al., 2022a,b). Due to difficulties of conducting random sampling during the pandemic, method of quota sampling was adopted to recruit participants based on two characteristics- faculty and year of study. Quota sampling method has been adopted in many research studies on psychology (Hungin et al., 2003; Nicolai et al., 2022). While it was criticized as having bias and difficulty in generalizability, it has the benefits of being cost-effective (Sharma, 2017). Besides, there are many studies that are based on a single university on the scientific literature (e.g., Mant et al., 2021; Fung et al., 2022; Yu et al., 2022).

We had obtained approval from the Institutional Review Board before we collected the data. Prior to responding to the online questionnaire, participants were informed of the purpose of the study and data confidentiality. The research team also obtained formal consent from each participant. After the survey, each student received a HK$100 supermarket coupon for appreciating his/her participation. A total of 1,648 participants (mean age = 20.09 ± 1.37) completed the survey. There were 696 (42.2%) males and 854 (51.8%) females (98 [5.9%] missing); 35 (2.1%) students indicated their student status as “international” (including those from mainland China) and 1,613 (97.9%) students reported their student status as “local.”

### 4.2. Measures

#### 4.2.1. Stress due to COVID-19 scale (COVID-19 stress scale)

Stress due to COVID-19 was assessed by a self-developed scale. This scale assesses three aspects with 15 items, including worries one may have about the pandemic-related danger and contamination (five items), the perceived pandemic-related socio-economic consequences (five items), and one’s checking behavior because of concerns about COVID-19 (five items), with reference to the work of Taylor et al. (2020). For each item, participants indicated their answers on a scale with five points (“0” = “Not at all” to “4” = “Always”). The composite score of this scale was calculated based on an average of all item scores. Table 1 shows the items in the scale.

**Table 1:** Items of the COVID-19 stress scale and the scale of stress due to “social event” (SEI).

Items of COVID-19 stress scale	
Danger and contamination	
1.	“I am worried about catching the virus”
2.	“I am worried that basic hygiene (e.g., handwashing) is not enough to keep me safe from the virus”
3.	“I am worried that people around me will infect me with the virus”
4.	“I am worried that if I touched something in a public space (e.g., handrail, door handle), I would catch the virus”
5.	“I am worried that if someone coughed or sneezed near me, I would catch the virus”
Socio-economic consequence	
1.	“I am worried about supermarkets running out of food or water”
2.	“I am worried about supermarkets or drug stores running out of cleaning or disinfectant supplies”
3.	“I am worried that online shops running out of supplies”
4.	“I am worried that online shops’ delivery will be delayed”
5.	“I am worried that the economy will collapse because of COVID-19”
Checking behavior	
1.	“Social media posts concerning COVID-19”
2.	“Seeking reassurance from friends or family about COVID-19”
3.	“Checking your own body for signs of infection (e.g., taking your temperature)”
4.	“Asking health professionals (e.g., doctors or pharmacists) for advice about COVID-19”
5.	“Searched the Internet for treatments for COVID-19”
**Items of SEI**	
Social event (Worries)	
1.	“I am worried about the social event”
2.	“I feel stressful about the social event”
3.	“I am worried that the Hong Kong Government will not be able to protect my loved one”
4.	“I am disturbed by the violence involved in the social event”
5.	“I am worried that our lives would be adversely affected by the social event”
6.	“I am worried that freedom in Hong Kong would diminish”
7.	“I am disappointed by the action taken by the Hong Kong Government to handle the social event”
Social event (Behavior Check)
8.	“Social media posts concerning the social events”
9.	“Discussion with friends on the social event”
10.	“Searched the Internet for news about the social event”

For both scales, each item is rated on a five-point scale: “0” = “Not at All,” “1” = “Rarely,” “2” = “Sometimes,” “3” = “Often,” and “4” = “Always.”

#### 4.2.2. Stress due to social event

Stress due to the “social event” in Hong Kong (SEI) was assessed by a self-developed scale. This 10-item measure includes two dimensions regarding one’s worries about the “social event” (seven items) and concerns about the “social event” (three items). For each item, participants indicated their answers on a scale with five points (“0” = “Not at all” to “4” = “Always”). The composite score of this scale was calculated based on an average of all item scores. The items of the scale are shown in Table 1.

#### 4.2.3. Depression (CESD-R)

Participants’ depressive symptoms were assessed by the “Centre for Epidemiological Studies Depression Scale Revised (CESD-R).” Through nine symptom categories, CESD-R measures depressive disorder based on the criteria defined in the “American Psychiatric Association Diagnostic and Statistical Manual of Mental Disorders, Fifth Edition (DSM-V)” (Radloff, 1977; Eaton et al., 2004). The construct was adopted in many previous studies and showed good psychometric properties (Van Dam and Earleywine, 2011; Ip et al., 2016; Li et al., 2019). For each item, students needed to report to what extent they feel/behave in the described way with a five-point scale (“0” = “Not at all or less than 1 day” to “4” = “Nearly every day for 2 weeks”). The composite score was the sum of scores of all items.

#### 4.2.4. Beliefs of adversity

Beliefs of adversity (BA) was examined by the “Chinese Cultural Beliefs about Adversity Scale” (Shek, 2004). This scale includes a total of nine items (seven items reflect positive traditional Chinese values related to adversity; two items reflect negative Chinese values related to adversity). These values are basically related to optimistic and hopeful beliefs about adversity. All the items were evaluated on a measure with six points (“1” = “Strongly disagree” to “6” = “Strongly agree”). The composite score was obtained by averaging all question scores (with the scores of the two items for negative beliefs being reversed).

#### 4.2.5. Psychosocial competence (resilience and emotional competence: REC)

Two subscales (resilience: three items; emotional competence: three items) in the “Chinese Positive Youth Development Scale (CPYDS)” (Shek et al., 2007) were used to assess resilience and emotional competence. For each question, each student was invited to report to what degree he/she agrees or does not agree with the statement on a measure with six points (“1” = “Strongly disagree” to “6” = “Strongly agree”). The composite score was obtained by averaging all question scores.

#### 4.2.6. Flourishing

University students’ Flourishing (FH) was measured by the “Flourishing Scale (FS)” comprised of eight items regarding a person’s self-perceived performance in multiple domains, including interpersonal relationship, meaning of life, healthy functioning, self-esteem, and so on (Diener et al., 2010; Li et al., 2020). For each item, one was required to report to what degree one agrees with the statement on a measure of six points (“1” = “Strongly disagree” to “7” = “Strongly agree”). The scale composite score was the mean of all question scores.

#### 4.2.7. Life satisfaction

Life satisfaction (LS) was assessed through the “Satisfaction With Life Scale (SWLS)” (Diener et al., 1985; Lin and Shek, 2019). The SWLS measures a person’s general satisfaction with life. It includes five items evaluated on a scale of six points (“1” = “Strongly disagree” to “6” = “Strongly agree”). The average score of all items was generated as the total score.

### 4.3. Data analysis plan

First, we computed mean score, standard deviation, Cronbach’s α coefficient, and mean inter-item correlation of each major variable. Then, separate multiple regression analyses were conducted to test the predictive effects of the COVID-19 Stress Scale, SEI, and the four positive psychological attributes on depression, with covariates (gender, student status [local vs. international], and age) being statistically controlled. Third, hierarchical multiple regression analyses were conducted to examine the moderating effect of positive psychological attributes on predictive effects of the COVID-19 Stress Scale and SEI on depression, respectively, with control of the covariates. Based on the conceptual framework that the four positive psychological attributes (BA, REC, FH, and LS) are foundational elements in positive psychology, we adopted the composite score of positive psychological attributes (POS; average of the mean scores of the four positive psychological attributes) as the moderator in the analyses. Bootstrapped bias-corrected (BC) 95% confidence intervals (CIs) for regression coefficients were also computed (2,000 re-samplings; Hayes, 2013).

## 5. Results

### 5.1. Descriptive profile, reliability, and correlations

Table 2 shows the descriptive profile and reliability of the variables covered in this study. The Cronbach’s α for different variables ranged between 0.73 and 0.96. The variable correlations are shown in Table 3. The COVID-19 Stress Scale (*r* = 0.35, *p* < 0.001) and SEI (*r* = 0.20, *p* < 0.001) were positively correlated with depression, while depression was negatively correlated with POS and all the individual positive attributes (*r*s = −0.41 to-0.26, *p*s < 0.001). Positive correlation existed among all positive attributes (*r*s = 0.30 to 0.69, *p*s < 0.001) and between positive attributes and POS (*r*s = 0.70 to 0.91, *p*s < 0.001). In addition, gender and student status were associated with most of the major variables (*r*s = −0.14 to 0.08, *p*s < 0.05) except for LS and COVID-19 Stress Scale. Age was significantly correlated with depression, LS and REC (*r*s = −0.08 to 0.06, *p*s < 0.05). We controlled for the effects of gender, student status, and age in the regression analyses. Given the relatively high correlations among positive variables, discrete multiple regression analyses were done in the next step to avoid multicollinearity.

**Table 2:** Mean (*M*), standard deviation (*SD*), Cronbach’s α, and mean inter-item correlation of variables.

	Female *n (%)*	Male *n (%)*		
Gender	854 (51.8)	696 (42.2)		
Student status	1,613 (97.6)	35 (2.1)		
	***M***	***SD***	**Cronbach’s α**	**Mean IIC**
Age	20.09	1.37		
Stress due to COVID-19	1.54	0.70	0.90	0.39
Stress due to social event	2.31	0.82	0.88	0.43
Depression	18.82	15.21	0.96	0.52
**Positive psychological factors**	3.94	0.68	0.84^a^	0.21
Resilience and emotional competence	4.00	0.78	0.86	0.51
Belief of adversity	3.89	0.65	0.73	0.24
Flourishing	4.50	1.01	0.91	0.57
Life satisfaction	3.38	0.94	0.87	0.58

Gender: 1 = female students, 0 = male students; Student status: 1 = local students, 0 = international students; IIC: inter-item correlation; ^a^the Cronbach’s *α* of positive psychological factors was the mean of the *α* coefficients of all positive variables given the different range of the scales.

**Table 3:** Correlations among variables.

	1	2	3	4	5	6	7	8	9	10	11
1. Gender	1										
2. Student status	0.02	1									
3. Age	0.05^*^	−0.13^***^	1								
4. C19S	−0.11^***^	0.02	−0.05	1							
5. SEI	−0.09^***^	0.08^**^	−0.004	0.42^***^	1						
6. Depression	−0.08^**^	0.08^***^	−0.08^***^	0.35^***^	0.20^***^	1					
**7. POS**	−0.06^*^	−0.14^***^	0.02	−0.05^*^	0.01	−0.41^***^	1				
8. REC	−0.01^**^	−0.13^***^	0.06^*^	−0.05^*^	0.08^**^	−0.38^***^	0.81^***^	1			
9. BA	−0.09^**^	−0.14^***^	0.03	0.01	0.02	−0.34^***^	0.70^***^	0.57^***^	1		
10. FH	−0.05^*^	−0.11^***^	0.03	−0.05^*^	0.04	−0.36^***^	0.91^***^	0.69^***^	0.53^***^	1	
11. LS	0.01	−0.07^**^	−0.06^*^	−0.06^*^	−0.11^***^	−0.26^***^	0.75^***^	0.38^***^	0.30^***^	0.60^***^	1

Gender: 1 = female students, 0 = male students; Student status: 1 = local students, 0 = international students; C19S: COVID-19 Stress Scale; SEI: stress due to social event; POS: composite positive psychological factors; REC: resilience and emotional competence; BA: beliefs of adversity; FH: flourishing; LS: life satisfaction; ^*^p < 0.05, ^**^p < 0.01, ^***^p < 0.001.

### 5.2. Multiple regression analyses

Table 4 shows the results of discrete multiple regression analyses of the predictive effects of COVID-19 Stress Scale, SEI, and the four positive psychological attributes on depression. After controlling for effects of gender, student status, and age, COVID-19 Stress and SEI significantly and positively predicted depression (COVID-19 Stress: *B* = 6.77, BC 95%CI = [5.61, 7.90], *β* = 0.31, *p* < 0.001, Cohen’s *f*^2^ = 0.091; SEI: *B* = 1.12, BC 95%CI = [0.20, 2.02], *β* = 0.06, *p* < 0.05, Cohen’s *f*^2^ = 0.003). All positive psychological factors, REC (*B* = −7.20, BC 95%CI = [−8.08, −6.36], *β* = −0.37, *p* < 0.001, Cohen’s *f*^2^ = 0.178), BA (*B* = −8.20, BC 95%CI = [−9.20, −7.23], *β* = −0.35, *p* < 0.001, Cohen’s *f*^2^ = 0.157), FH (*B* = −5.30, BC 95%CI = [−5.93, −4.67], *β* = −0.35, *p* < 0.001, Cohen’s *f*^2^ = 0.160), and LS (*B* = −3.75, BC 95%CI = [−4.47, −3.06], *β* = −0.23, *p* < 0.001, Cohen’s *f*^2^ = 0.065), significantly and negatively predicted depression.

**Table 4:** Discrete multiple regression analyses for the predicting effects of COVID-19 stress and stress due to social event and positive psychological factors on depression.

	Model 2^a^		Model 3			Model 4			Model 5				Model 6		
	*B* (BC 95% CI)	*β*	*f* ^2^	*B* (BC 95% CI)	*β*	*f* ^2^	*B* (BC 95% CI)	*β*	*f* ^2^	*B* (BC 95% CI)	*β*	*f* ^2^	*B* (BC 95% CI)	*β*	*f* ^2^
**Control**															
Age	−0.63 (−1.09, −0.18)	−0.06^*^	0.004	−0.45 (−0.90, −0.03)	−0.04	0.002	−0.55 (−0.98, −0.10)	−0.05^*^	0.003	−0.57 (−1.01, −0.15)	−0.05^*^	0.003	−0.80 (−1.26, −0.32)	−0.07^**^	0.006
Gender	−0.00 (−0.01, −0.00)	−0.04	0.002	−0.00 (−0.01, −0.00)	−0.06^**^	0.005	−0.00 (−0.01, −0.00)	−0.07^**^	0.006	−0.00 (−0.01, −0.00)	−0.06^*^	0.004	−0.11 (−0.01, −0.00)	−0.04	0.002
Student status	6.58 (3.40, 9.50)	0.06^**^	0.004	1.31 (−2.64, 5.09)	0.01	0.000	1.68 (−2.07, 5.06)	0.02	0.000	2.50 (−1.03, 5.64)	0.02	0.001	4.86 (1.33, 8.01)	0.05^*^	0.003
**Predictors**															
C19S	6.77 (5.61, 7.90)	0.31^***^	0.091	5.89 (4.83, 6.99)	0.27^***^	0.080	6.72 (5.75, 7.74)	0.31^***^	0.104	6.05 (5.05, 7.10)	0.28^***^	0.084	6.65 (5.55, 7.73)	0.31^***^	0.093
SEI	1.12 (0.20, 2.02)	0.06^*^	0.003	2.01 (1.21, 2.81)	0.11^***^	0.013	1.28 (0.41, 2.08)	0.07^**^	0.005	1.68 (0.83, 2.56)	0.09^***^	0.009	0.73 (−0.15, 1.60)	0.04	0.002
REC				−7.20 (−8.08, −6.36)	−0.37^***^	0.178									
BA							−8.20 (−9.20, −7.23)	−0.35^***^	0.157						
FH										−5.30 (−5.93, −4.67)	−0.35^***^	0.160			
LS													−3.75 (−4.47, −3.06)	−0.23^***^	0.065
*F* change ^b^	108.53^***^			182.74^***^			169.58^***^			171.59^***^			112.59^***^		
*R*^2^ change ^b^	0.115			0.246			0.233			0.235			0.168		

a Model 1 (for control variables only) is omitted here to keep the size of the table.

b Model 2 vs. Model 1 (omitted), Models 3–6 vs. Model 2; Gender: 1 = female students, 0 = male students; Student status: 1 = local students, 0 = international students. C19S: COVID-19 Stress Scale; SEI: stress due to social event; REC: resilience and emotional competence; BA: beliefs of adversity; FH: flourishing; LS: life satisfaction; ^*^p < 0.05, ^**^p < 0.01, ^***^p < 0.001.

**Figure 1:**
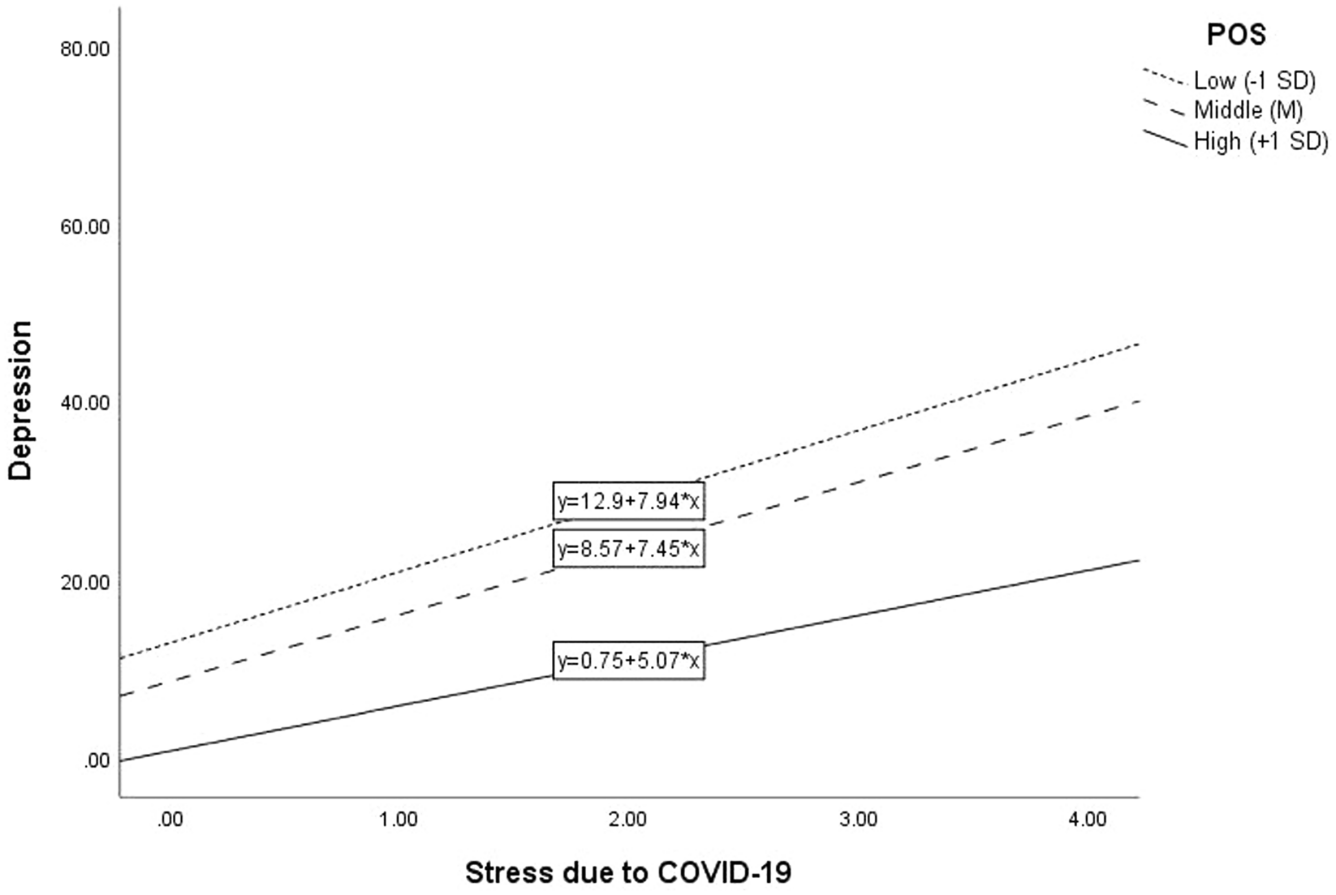
Moderating effect of composite positive psychological factors (POS) on the relationship between COVID-19 Stress and depression.

Table 5 shows the results of multiple regression analyses of the predicting effects of COVID-19 stress, POS, and their interaction on depression. After controlling for effects of gender, student status, and age, COVID-19 Stress significantly and positively predicted depression (*B* = 6.82, BC 95%CI = [5.92, 7.74], *β* = 0.31, *p* < 0.001, Cohen’s *f*^2^ = 0.135), and POS negatively predicted depression (*B* = −8.89, BC 95%CI = [−9.95, −7.89], *β* = −0.40, *p* < 0.001, Cohen’s *f*^2^ = 0.214). The predicting effect of the interaction between COVID-19 stress and POS was significant (*B* = −0.77, BC 95%CI = [−1.36, −0.16], *β* = −0.06, *p* < 0.01, Cohen’s *f*^2^ = 0.005), indicating that the predicting effect of COVID-19 stress on depression was moderated by POS. Specifically, the predictive effect of COVID-19 stress on depression was stronger among the participants with lower POS (−1 SD; *B* = 7.94, BC 95%CI = [5.25, 10.50], *p* < 0.001) than that among the participants with higher POS (+1 SD; *B* = 5.07, BC 95%CI = [3.43, 6.83], *p* < 0.001). Figure 1 shows the moderating effect graphically.

**Table 5:** Multiple regression analyses for the moderating effect of composite positive psychological factors (POS) on the relationship between COVID-19 Stress and depression.

Predictor	*B*	*BC 95% CI*		*SE*	β	*t*	Cohen’s *f*^2^	*F* change	*R^2^* change
		Lower	Upper						
**Step 1**									
Age	−0.75	−1.27	−0.25	0.26	−0.07	−2.74^**^	0.005	9.54^***^	0.017
Gender	−0.01	−0.01	−0.00	0.00	−0.08	−3.13^**^	0.006		
Student status	7.55	3.84	10.91	1.73	0.07	2.90^**^	0.005		
**Step 2**									
C19S	6.82	5.92	7.74	0.46	0.31	14.88^***^	0.135	303.23^***^	0.265
POS	−8.89	−9.95	−7.89	0.51	−0.40	−18.73^***^	0.214		
**Step 3**									
C19S × POS	−0.77	−1.36	−0.16	0.32	−0.06	−2.75 ^**^	0.005	7.58^**^	0.003

Gender: 1 = female students, 0 = male students; Student status: 1 = local students, 0 = international students. C19S: COVID-19 Stress Scale; POS: positive psychological factors; ^**^p < 0.01, ^***^p < 0.001.

**Figure 2:**
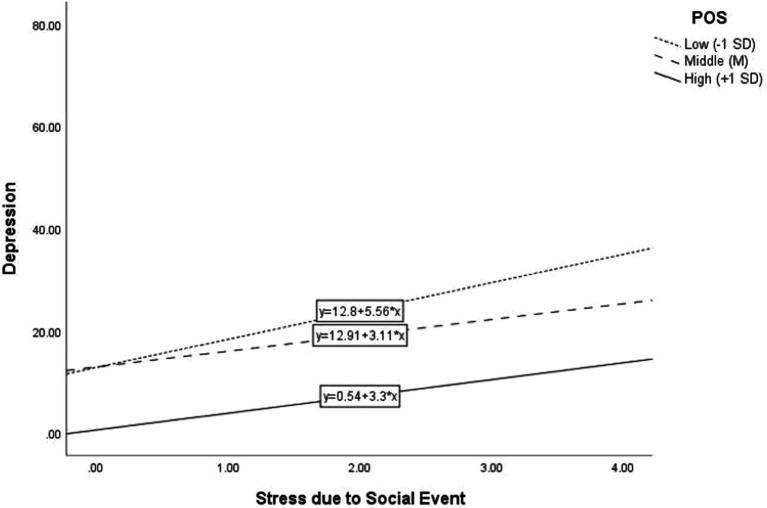
Moderating effect of composite positive psychological factors (POS) on the relationship between stress due to social event and depression.

Table 6 shows the results of multiple regression analyses of the predictive effects of SEI, POS, and their interaction on depression. After controlling for effects of gender, student status, and age, SEI positively predicted depression (*B* = 3.62, BC 95%CI = [2.76, 4.38], *β* = 0.20, *p* < 0.001, Cohen’s *f*^2^ = 0.049), and POS negatively predicted depression (*B* = −9.35, BC 95%CI = [−10.35, −8.41], *β* = −0.42, *p* < 0.001, Cohen’s *f*^2^ = 0.219). The predicting effect of the interaction between SEI and POS was significant (*B* = −0.89, BC 95%CI = [−1.46, −0.20], *β* = −0.07, *p* < 0.01, Cohen’s *f*^2^ = 0.006). This indicates that the predictive effect of SEI on depression was moderated by POS. Specifically, the predicting effect of SEI on depression was stronger among participants with lower POS (−1 SD; *B* = 5.56, BC 95%CI = [3.63, 7.54], *p* < 0.001) than that among participants with higher POS (+1 SD; *B* = 3.30, BC 95%CI = [2.13, 4.53], *p* < 0.001). Figure 2 shows the moderating effect graphically.

**Table 6:** Multiple regression analyses for the moderating effect of composite positive psychological factors (POS) on the relationship between stress due to social event (SEI) and depression.

Predictor	*B*	*BC 95% CI*		*SE*	β	*t*	Cohen’s *f*^2^	*F* change	*R^2^* change
		Lower	Upper						
**Step 1**									
Age	−0.75	−1.27	−0.21	0.26	−0.07	−2.74^**^	0.005	9.54^***^	0.017
Gender	−0.01	−0.01	−0.00	0.00	−0.08	−3.13^**^	0.006		
Student status	7.55	3.68	10.81	1.75	0.07	2.90^**^	0.005		
**Step 2**									
SEI	3.62	2.76	4.38	0.40	0.20	8.93^***^	0.049	217.88^***^	0.206
POS	−9.35	−10.35	−8.41	0.51	−0.42	−18.98^***^	0.219		
**Step 3**									
SEI × POS	−0.89	−1.46	−0.20	0.31	−0.07	−3.17 ^**^	0.006	10.04^**^	0.005

Gender: 1 = female students, 0 = male students; Student status: 1 = local students, 0 = international students. SEI: stress due to social event; POS: positive psychological factors; ^**^p < 0.01, ^***^p < 0.001.

## 6. Discussion

There are few studies on university students’ mental health and its risk and protective factors in Hong Kong during the COVID-19 pandemic. The present study investigated the predictive role of stresses related to the pandemic and the “social event” in depression in Hong Kong university students and the moderating function of positive psychological factors in the stress-mental health relationships. The study has several conceptual advances and unique contributions. First, while the existing literature mainly focuses on the association between pandemic-related stress and mental health problems such as depression, there are few studies investigating the predictive effects of stresses from both the pandemic and the “social event.” In fact, Hong Kong has not only experienced (and is experiencing) the event of pandemic, Hong Kong people has also undergone the “social event” and its aftermath. Hence, we should understand the impact of such stresses on student mental health. Second, while some studies (e.g., Wong et al., 2021) examined both stresses from the pandemic and the “social event” on mental health, they mainly focused on adults and community samples but not university students. Besides, these studies did not examine the moderating effect of positive psychological attributes on the stress-mental health relationship. Third, instead of focusing on one or two positive psychological attributes, we used a set of positive psychological constructs, including beliefs of adversity, psychosocial competence, flourishing, and life satisfaction.

Methodologically, there are several strengths of this study. First, it employed a large sample size that would yield more reliable results. Second, as many existing studies were conducted in Western societies, there is a need to understand the situation and examine the hypothesized relationship in non-Western contexts. By focusing on Hong Kong university students, the present study contributes to existing literature in a non-Western context.

Results of the present study support Hypotheses 1a and 1b, suggesting that students’ stresses related to COVID-19 and the “social event” positively predicted depression. This observation is in line with the existing literature arguing that COVID-19-related stress or stressors were associated with increased depression in adolescents (Ellis et al., 2020) and university students (Rudenstine et al., 2021; Lu et al., 2022). However, it is noteworthy that there are inconsistent findings in the literature. For example, a two-wave study on emerging adults showed that baseline pandemic-related stress did not predict the change in depression over time (Kujawa et al., 2020). Besides, while the predictive role of stress on depression has been well established in existing literature (Hammen, 2015), whether the pandemic-related stress also predicts depression has not been adequately studied. Thus, findings from this study provide further support to the positive association between COVID-19-related stress and depression in university students.

Regarding the association between social-unrest-related stress and depression, there are few existing studies as well as inconsistent findings. Wong et al. (2021)’s study on Hong Kong community members showed that depressive symptoms were predicted by pandemic-related stress but not social-unrest-related stress. However, the two studies conducted by Hou W. K. et al. (2021, 2022) based on large samples of Hong Kong residents showed both pandemic-related and social-unrest-related stresses predicted higher depression. In view of the presence of the “social event” and the pandemic in Hong Kong in recent years, the present study provides significant empirical evidence for the positive predictive role of both stresses on depression in Hong Kong university students.

Findings of this study support Hypothesis 2, suggesting negative predictive relationships between positive psychological factors and depression. Specifically, findings revealed that beliefs of adversity, resilience and emotional competence, flourishing, and life satisfaction negatively predicted depression in university students during the pandemic. The findings extend and contribute to the existing literature. Firstly, the findings add further evidence to the existing literature on the negative predictive effect of life satisfaction, resilience, and emotional competence on depression. For example, both cross-sectional and longitudinal studies showed that life satisfaction negatively predicted depression in university students, including the studies conducted during the pandemic (e.g., Dorahy et al., 2000; Hartstone and Medvedev, 2021; Lopes and Nihei, 2021). Other studies showed that higher resilience would predict lower depression in university students (Kapikiran and Acun-Kapikiran, 2016; Mak et al., 2018). Also, studies showed that emotional competence negatively predicted depression (Kwok and Gu, 2017; Elsina and Martinsone, 2021).

Second, the findings extend the existing literature on the negative predictive role of flourishing and beliefs of adversity in depression. While the concept of flourishing has been promoted in positive psychology in recent years, few empirical studies examined its relationship with mental health problems in university students, particularly in the Chinese contexts. One study showed that flourishing negatively predicted depression in university students in Iran (Soleimani et al., 2015). Another study showed that lower flourishing predicted higher depression in adolescent bullying victims (Rey et al., 2019). Findings from this study extend the existing literature on the negative predictive role of flourishing in depression. Regarding beliefs of adversity, it refers to individuals’ positive values and beliefs about adversity that would bring positive influence on their behaviors in coping with adversity (Huang et al., 2014). Based on such work, it is argued that beliefs of adversity would protect individuals from mental health problems. However, there is quite limited literature on the association between beliefs about adversity and mental health problems in university students. Therefore, findings of the study provide evidence for the negative predicting role of beliefs about adversity in depression in university students.

Supporting Hypotheses 3a and 3b, the present study showed that the predictive effects of interaction between COVID-19 stress and “social event” stress and positive psychological factors on depression were significant although the effect size was small. This suggests that positive psychological factors moderated the positive association between stresses and depression. The findings are in line with the existing literature on the moderating role of specific positive psychological factors. For example, a study on Hong Kong primary school students showed that life satisfaction moderated the association between parental anxiety and student depression (Tam et al., 2018). Also, a recent research study based on 1,718 students in one university in Australia showed that the students’ resilience moderated the association between social isolation and depression during the pandemic (Liu et al., 2021).

However, there have been inconclusive findings regarding the moderating role of certain positive psychological factors. For example, while some studies showed that emotional competence could moderate the linkage between adverse life events and mental illness, such as suicidal ideation (Cha and Nock, 2009; Kwok, 2014), there were also studies showing that emotional competence did not moderate the relationship between adverse life events and suicidal ideation (Wang et al., 2011). As Chinese culture highlights “emotional restraint” or even “emotional suppression” (Kwok, 2014), whether emotional competence (including emotional expression) could moderate the association between adverse life events/stress and mental illness should be further investigated. The study contributes to this part of literature by showing the moderating role of positive psychological attributes in the stress-depression relationship.

Theoretically, this study contributes to the generalizability of the positive youth development (PYD) theory, which was originated in Western cultures, in the Chinese context. For example, Shek et al. (2021b) revealed that PYD attributes moderated the relationship between the pandemic-related perceived threats and post-traumatic stress. Shek et al. (2022b) showed that PYD variables moderated the relationship between needs satisfaction and depression in university students. Besides, Kapikiran and Acun-Kapikiran (2016) showed that university students with higher resilience demonstrated better adaptation to adverse situations, which would protect them from mental health problems such as depression. The present findings are important responses to the reflections raised by Shek (2021) and Shek et al. (in press). Waters et al. (2022) also highlighted several positive psychological attributes that could help people to cope with stress during the pandemic. The featured PYD attributes included life meaning, coping style, self-compassion, courage to face adversity, gratitude, character strengths, positive affect, positive relationship processes, and positive connections. The present findings concur with such emphases.

Practically, the study contributes to the development of effective intervention programs for reducing students’ depression under stresses during the pandemic. It highlights the importance of PYD programs in promoting students’ mental health during the pandemic. For example, Arslan and Burke (2021) stressed the importance of positive psychology when students resume normal classes. With reference to Hong Kong, despite the emphases of different stakeholders on the need of psychosocial competence and positive psychological development in students, such coverage is very thin in the formal curriculum (Shek et al., 2021a). Obviously, there is a need to step up education on positive psychology in the higher education sector.

Although this study is pioneering in the area, several limitations should be noted. First, as cross-sectional data were collected in this study, we are not able to infer the causal relationships among the variables. As such, we should conduct longitudinal studies in the future. Second, we collected data *via* self-reported measures which might have response bias (Furnham and Henderson, 1982). Future studies should include data collected from the significant other of research participants to give a more triangulated picture. Third, while quota sampling has benefits of cost-effectiveness and creating a sample that illustrate the proportion of required groups based on participants’ characteristics, it has limitations of creating bias and representativeness (Iliyasu and Etikan, 2021). If possible, future studies should use random sampling strategies. Of course, in view of city lockdown and suspension of schools, it may be difficult, if not impossible to collect data using random sampling during the pandemic.

## Data availability statement

The raw data supporting the conclusions of this article will be made available by the authors, without undue reservation.

## Ethics statement

The studies involving human participants were reviewed and approved by the Institutional Review Board (or its Delegate) at the Hong Kong Polytechnic University. The patients/participants provided their written informed consent to participate in this study.

## Author contributions

DS obtained the research grant, conceived the research, contributed to all stages of the research work, and critically revised all versions of the manuscript. W-yC checked data analyses, drafted discussion section, and revised the whole manuscript. TW drafted the introduction section, conducted revision, and checked the manuscript. KZ conducted data analyses and drafted method and results sections. All authors contributed to the article and approved the submitted version.

## Funding

This study was financially supported by the University Grants Committee of Hong Kong, Wofoo Foundation, and the Research Matching Grant of the Research Grants Council.

## Conflict of interest

The authors declare that the research was conducted in the absence of any commercial or financial relationships that could be construed as a potential conflict of interest.

## Publisher’s note

All claims expressed in this article are solely those of the authors and do not necessarily represent those of their affiliated organizations, or those of the publisher, the editors and the reviewers. Any product that may be evaluated in this article, or claim that may be made by its manufacturer, is not guaranteed or endorsed by the publisher.
